# Supplementary material for: The first occurrence of machimosaurid crocodylomorphs from the Oxfordian of south-central Poland provides new insights into the distribution of macrophagous teleosauroids
Source: PeerJ. 2024 Mar 28;12:e17153. doi: 10.7717/peerj.17153 (PMC10981889; doi:10.7717/peerj.17153)
Supplement: Supplemental Information 1 [file peerj-12-17153-s001.docx]

| **Continuous** | C1 – apicobasal crown height  C2 – crown ratio  C3 – labial/lingual curvature  C4 – crown angle |
| --- | --- |
| **Discrete** | **General**  D1 – labial-lingual compression  D12 – shape of the crown apex  D13 – non procumbent or procumbent dentition |
|  | **Ornamentation**  D6 – presence of the anastomosed pattern  D7 – enamel ornamentation, lingual face  D8– enamel ornamentation, labial face  D9 – apicobasal ridges, relief  D11 – texture of enamel |
|  | **Carinae and/or serrations**  D2 – presence and size of true denticles  D3 – presence or absence of functionally serrated edges  D4 – denticle distribution  D10 – presence of ‘pseudodenticles’ |

**Discrete and continuous characters (taken from Johnson et al., 2022a) used in tooth morphometry of MZ VIII Vr-72**

**Measurements for continuous characters:**

| **Teeth number** | **Apicobasal (AB) crown height** | **Mesiodistal (MD) length** | **Labiolingual (LL) length** | **Tooth curvature outer** | **Tooth curvature inner** |
| --- | --- | --- | --- | --- | --- |
| **T1** | **16,47 mm** | **7 mm** | **6.60 mm** | **16.82 mm** | **13.14 mm** |
| **T2** | **13.65 mm** | **6.80 mm** | **6.76 mm** | **14. 10 mm** | **12.21 mm** |
| **T3** | **14.7 mm** | **7.20 mm** | **6.94 mm** | **14.81 mm** | **13.51 mm** |

**Avg. T1-T3: AB height: 14.94 mm; MD length: 7mm; LL length: 6,76 mm; Labiolingual curvature: 1.19.**

**Discrete characters:**

**D1:** Labial-lingual compression. Labial-lingual compression of the tooth: absent (0), weakly compressed (1) or strongly compressed (2). Modified from Young et al., 2016, Ch.184.

(0).Absent

**D2:** Presence and size of true denticles: absent (0), incipient microziphodonty (1), conspicuous microziphodonty (2) or macroziphodonty (3). Note, the terms ‘microziphodonty’ and ‘macroziphodonty’ follows that of Foffa et al. (2017). Modified from Young et al. 2016, Ch. 215.

(0).Absent

**D3:** Presence or absence of functionally serrated edges: absent (0) or present (1).

(1).Present

**D4:** Denticle distribution: non-contiguous (0) or contiguous (1). Modified from Young et al. 2016, Ch.216.

(0).Absent

**D5:** Presence of ‘pseudodenticles’: absent (0) or present (1).

(0).Absent

**D6:** Presence of anastomosed pattern: absent (0) or present (1). Anastomosed pattern refer to detailed, branching crenulations of enamel ridges on the apex of the tooth. Modified from Young et al. (2016), Ch.221.

(1).Present

**D7:** Enamel ornamentation, lingual side: absent (0), largely absent, or present with weak apicobasal ridges (1), present and consists of numerous, spaced defined apicobasal ridges (2) or present and consists of conspicuous, numerous, well-defined, closely packed apicobasal ridges (3). Modified from Foffa et al. (2018).

(3).Present and consists of conspicuous, numerous, well-defined, closely packed apicobasal ridges

**D8:** Enamel ornamentation, labial side: largely absent, or present with weak apicobasal ridges (0), present and consists of numerous, spaced defined apicobasal ridges (1) or present and consists of conspicuous, numerous, well-defined, closely packed apicobasal ridges (2). Modified from Foffa et al. (2018).

(2).Present and consists of numerous, tightly-spaced defined apicobasal ridges

**D9:** Enamel ridges, relief: absent or extremely low (absent macroscopically) (0), low relief but macroscopically distinct, <0.5 mm (1) or medium relief, <1 mm (2). Modified from Foffa et al., 2018.

(2).Medium relief <1mm

**D10:** Presence or absence of false denticles: absent (0) or present (1). Denticles refer to ornamentation that interferes with the carinae. Modified from Young et al. 2016, Ch.214.

(0).Absent

**D11:** Texture of enamel: smooth (0) or pebbled (1).

(0).Smooth

**D12:** Shape of tooth crown apex: sharp and pointed (0) or blunt and round (1). Modified from Young et al. 2016, Ch.206.

(0).Sharp and pointed

**D13:** Non-procumbent or procumbent dentition: non-procumbent (0) or procumbent (1).

(0).Non-procumbent
