# Supplementary material for: The first occurrence of machimosaurid crocodylomorphs from the Oxfordian of south-central Poland provides new insights into the distribution of macrophagous teleosauroids
Source: PeerJ. 2024 Mar 28;12:e17153. doi: 10.7717/peerj.17153 (PMC10981889; doi:10.7717/peerj.17153)

**Supplementary material 3**

**Eigenvalues and PCoA results for Johnson et al. (2022) teleosauroid taxa with MZ VIII Vr-72 integrated into dataset (using Madzia et al. 2021 methodology)**

| **Axis** | **Eigenvalue** | **Percent** |
| --- | --- | --- |
| **1** | **0.037244** | **37.997** |
| **2** | **0.010072** | **10.275** |
| **3** | **0.0051438** | **5.2476** |
| **4** | **0.0030253** | **3.0864** |
| **5** | **0.0023337** | **2.3808** |
| **6** | **0.0016199** | **1.6526** |
| **7** | **0.0011004** | **1.1226** |
| **8** | **0.0010191** | **1.0397** |
| **9** | **0.00063357** | **0.64637** |
| **10** | **0.00041284** | **0.42118** |
| **11** | **0.00034338** | **0.35032** |
| **12** | **0.00020446** | **0.20859** |
| **13** | **0.00019188** | **0.19576** |
| **14** | **0.00014935** | **0.15236** |
| **15** | **5.8054E-05** | **0.059227** |
| **16** | **1.5159E-05** | **0.015465** |
| **17** | **8.3459E-06** | **0.0085145** |
| **18** | **1.7649E-06** | **0.0018005** |
| **19** | **4.7446E-19** | **4.8404E-16** |
| **20** | **-7.0142E-18** | **-7.1559E-15** |
| **21** | **-1.936E-06** | **-0.0019751** |
| **22** | **-2.9222E-06** | **-0.0029812** |
| **23** | **-2.6284E-05** | **-0.026815** |
| **24** | **-2.9983E-05** | **-0.030589** |
| **25** | **-4.6513E-05** | **-0.047452** |
| **26** | **-0.00014493** | **-0.14785** |
| **27** | **-0.00019483** | **-0.19876** |
| **28** | **-0.00020176** | **-0.20583** |
| **29** | **-0.00034387** | **-0.35081** |
| **30** | **-0.00039452** | **-0.40249** |


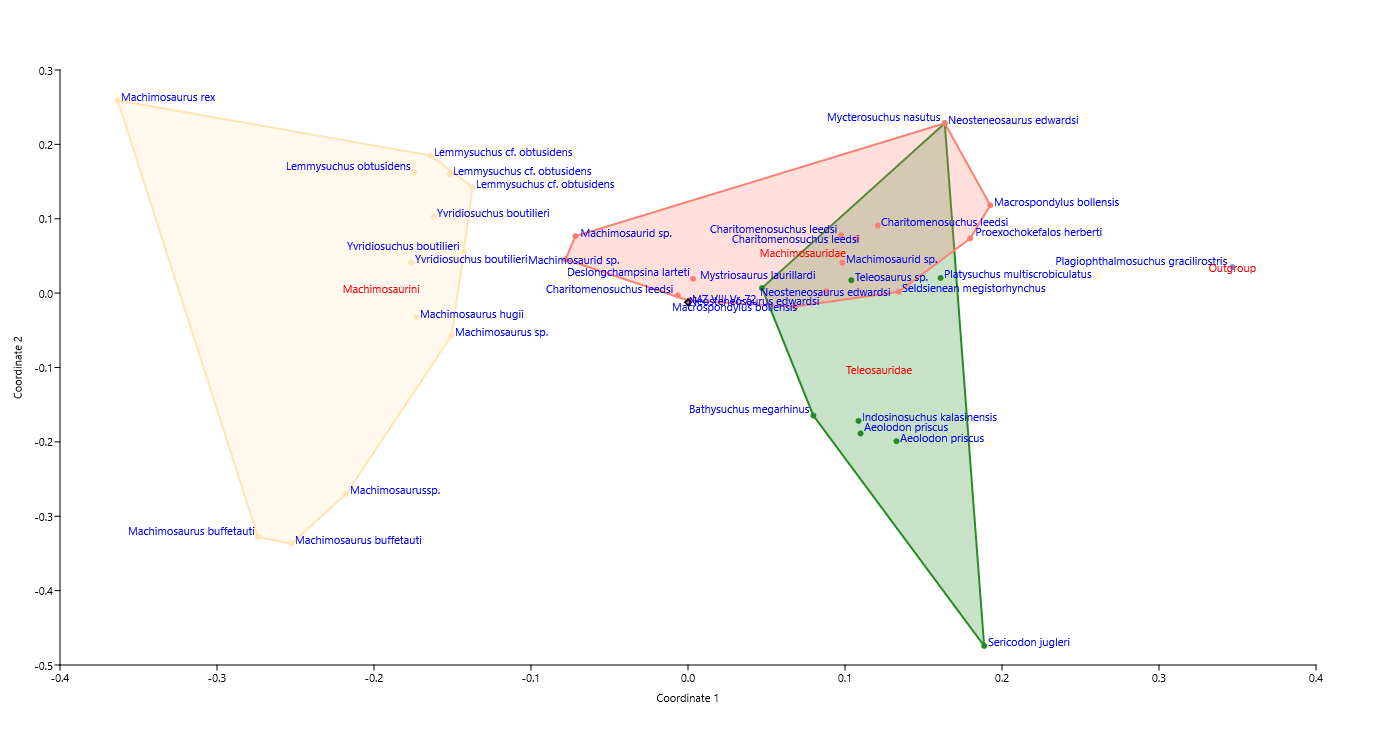


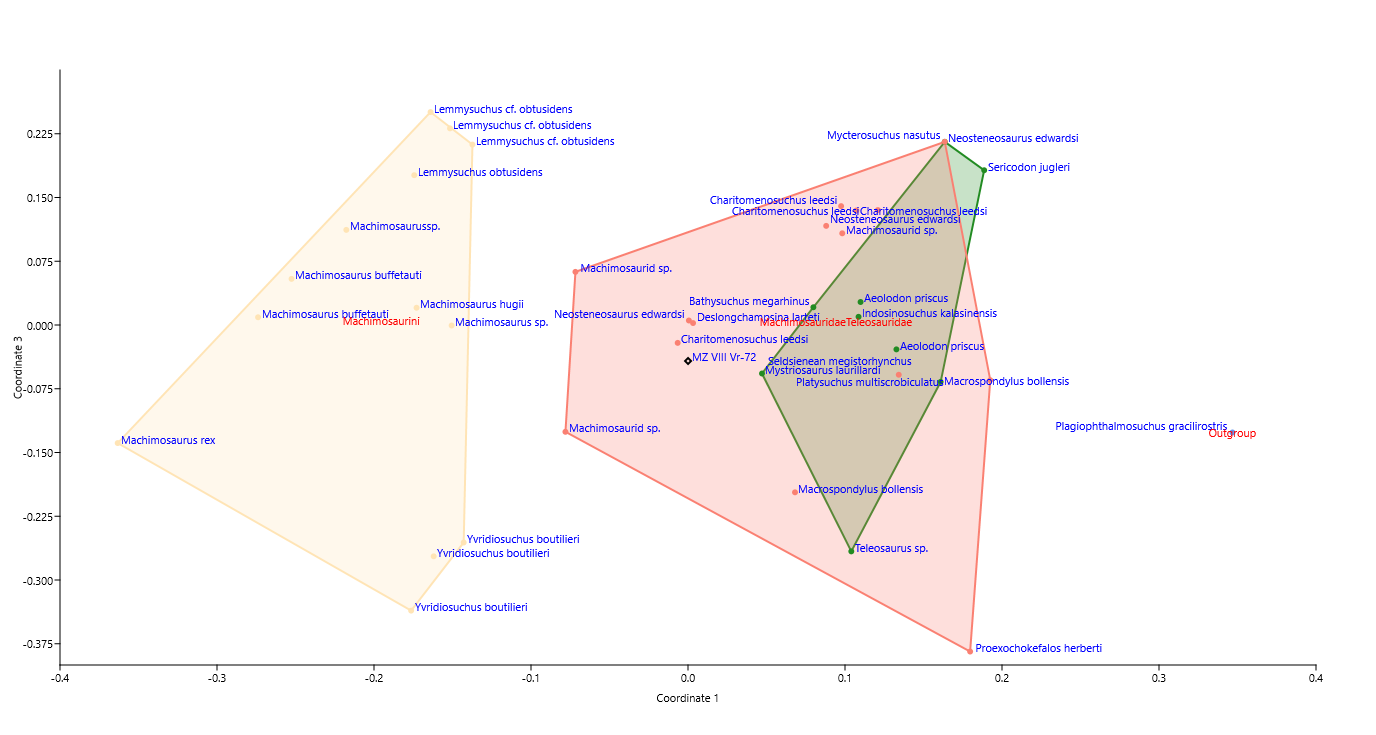

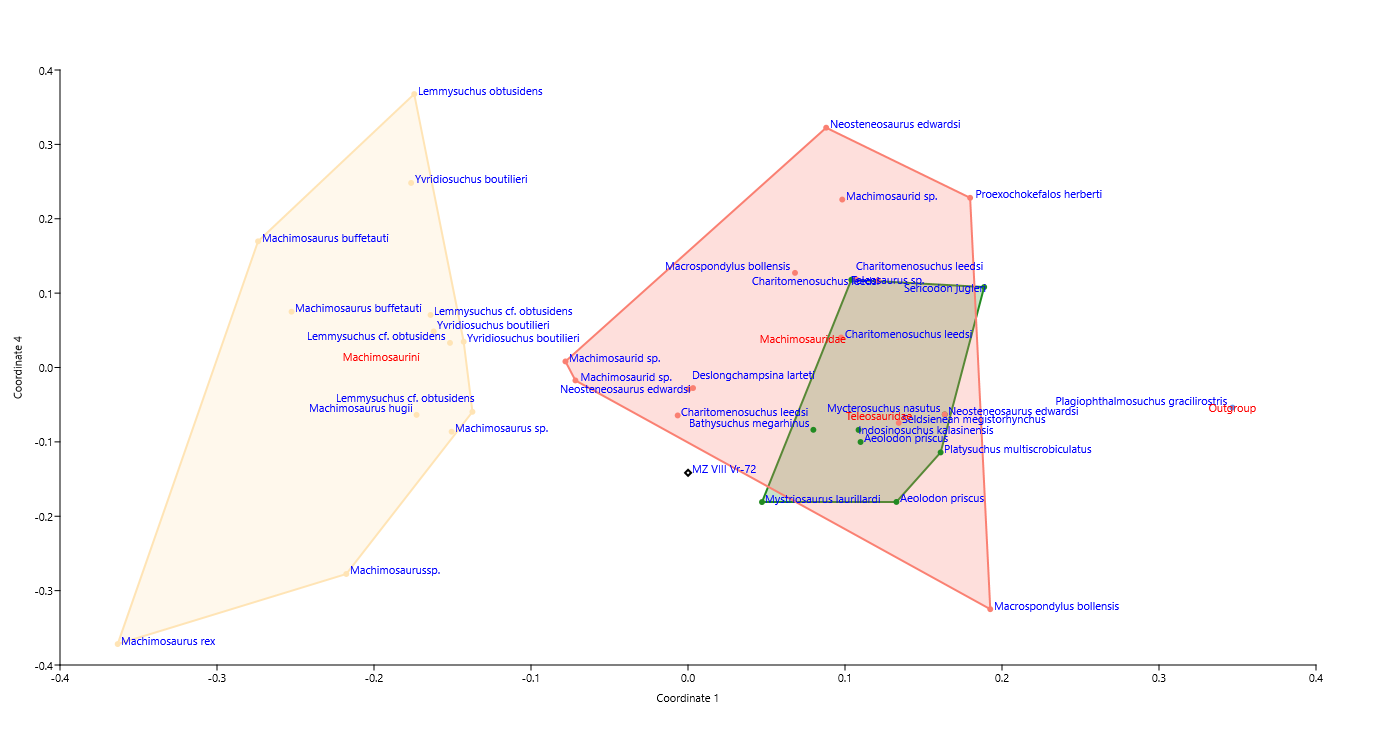

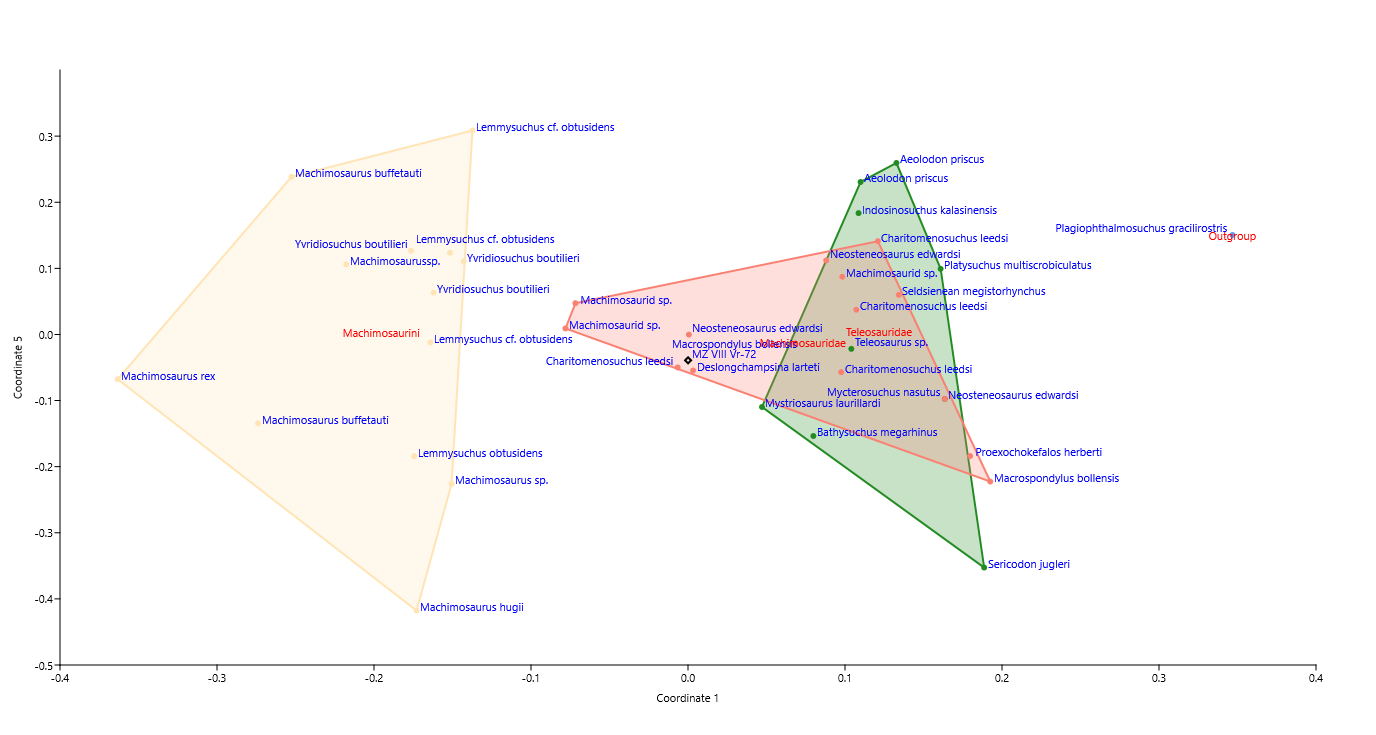

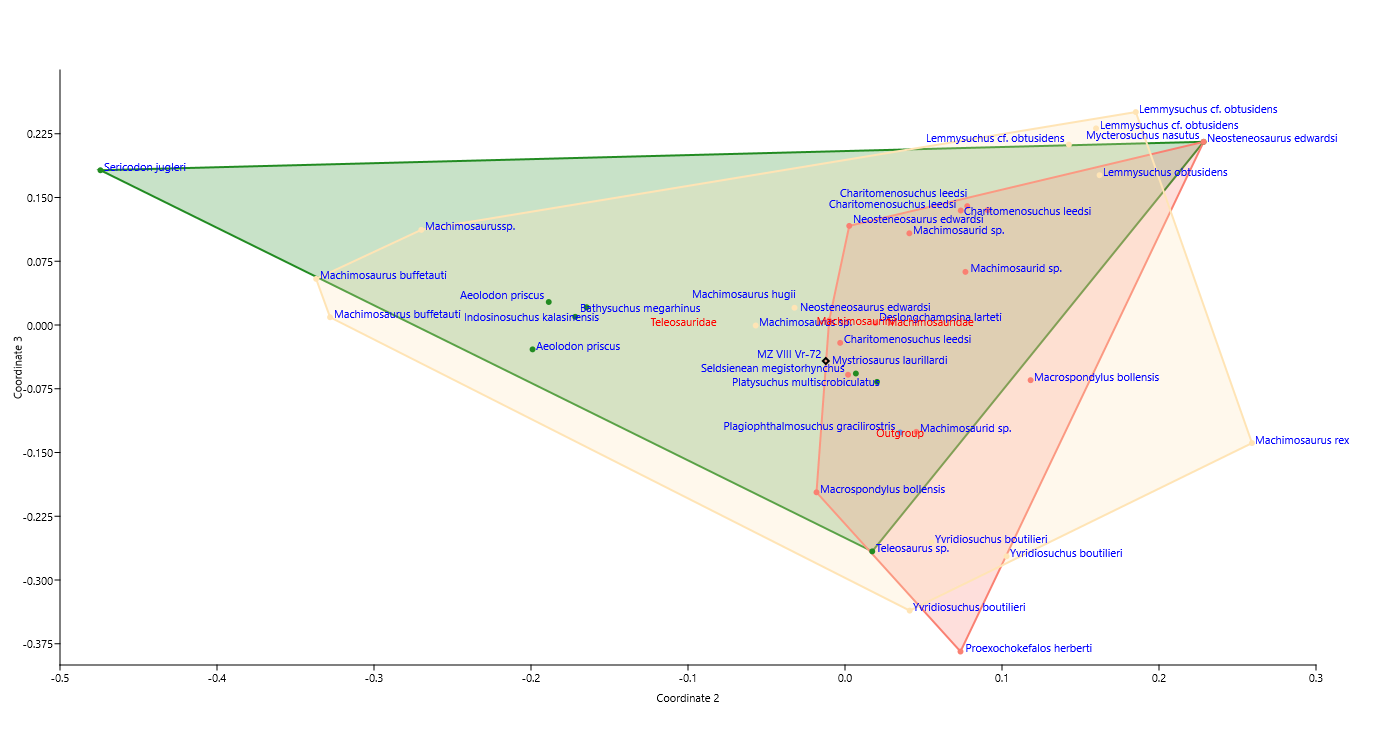

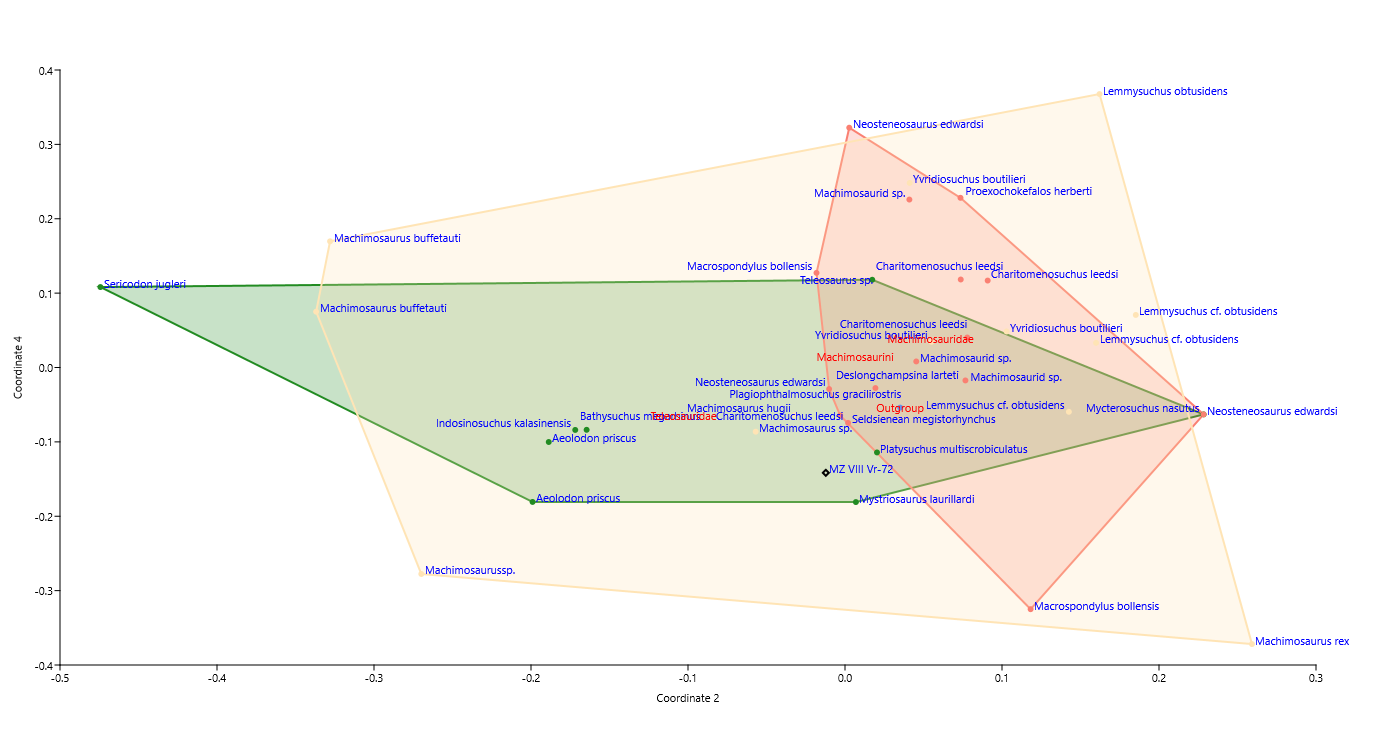

Supplement: Supplemental Information 3 [file peerj-12-17153-s003.docx]
